# Cytotoxic T-Lymphocyte-Associated Protein 4 Haploinsufficiency-Associated Inflammation Can Occur Independently of T-Cell Hyperproliferation

**DOI:** 10.3389/fimmu.2018.01715

**Published:** 2018-07-24

**Authors:** Carole Le Coz, Brian E. Nolan, Melissa Trofa, Alicia M. Kamsheh, Mustafa K. Khokha, Saquib A. Lakhani, Antonio Novelli, Elaine H. Zackai, Kathleen E. Sullivan, Silvana Briuglia, Tricia R. Bhatti, Neil Romberg

**Affiliations:** ^1^Division of Immunology and Allergy, The Children’s Hospital of Philadelphia, Philadelphia, PA, United States; ^2^Division of Rheumatology, The Children’s Hospital of Philadelphia, Philadelphia, PA, United States; ^3^Department of Genetics, Yale University School of Medicine, New Haven, CT, United States; ^4^Department of Pediatrics, Yale University School of Medicine, New Haven, CT, United States; ^5^The Pediatric Genomics Discovery Program, Yale University School of Medicine, New Haven, CT, United States; ^6^Laboratory of Molecular Genetics, Bambino Gesù Children’s Hospital, IRCCS, Rome, Italy; ^7^Division of Human Genetics, The Children’s Hospital of Philadelphia, Philadelphia, PA, United States; ^8^Department of Pediatrics, The Perelman School of Medicine at the University of Pennsylvania, Philadelphia, PA, United States; ^9^Department of Biomedical Science, University of Messina, Messina, Italy; ^10^Division of Pathology and Laboratory Medicine, The Children’s Hospital of Philadelphia, Philadelphia, PA, United States

**Keywords:** cytotoxic T-lymphocyte-associated protein 4, CD28, type 3 innate lymphoid cell, inflammation, regulatory T cell

## Abstract

Located contiguously on the long arm of the second chromosome are gene paralogs encoding the immunoglobulin-family co-activation receptors CD28 and cytotoxic T-lymphocyte-associated protein 4 (CTLA4). CD28 and CTLA4 share the same B7 ligands yet each provides opposing proliferative signals to T cells. Herein, we describe for the first time two unrelated subjects with coexisting CD28 and CTLA4 haploinsufficiency due to heterozygous microdeletions of chromosome 2q. Although their clinical phenotype, multi-organ inflammatory disease, is superficially similar to that of CTLA4 haploinsufficient autoimmune lymphoproliferative syndrome type V (ALPS5) patients, we demonstrate our subjects’ underlying immunopathology to be distinct. Unlike ALPS5 T cells which hyperproliferate to T-cell receptor-mediated activation and infiltrate organs, T cells from our subjects are hypoproliferative and do not. Instead of T cell infiltrates, biopsies of affected subject tissues demonstrated infiltrates of lineage negative lymphoid cells. This histologic feature correlated with significant increases in circulating type 3 innate lymphoid cells (ILC3s) and ILC3 cytokines, interleukin 22, and interleukin-17A. CTLA4-Ig monotherapy, which we trialed in one subject, was remarkably effective in controlling inflammatory diseases, normalizing ILC3 frequencies, and reducing ILC3 cytokine concentrations.

## Background

Located contiguously on the long arm of the second chromosome are gene paralogs encoding CD28 and cytotoxic T-lymphocyte-associated protein 4 (CTLA4). Both are immunoglobulin-family co-activation receptors that share the same B7 ligands yet each provides opposing proliferative signals to T cells (1). CD28 is a potent T-cell receptor (TCR) costimulatory molecule, whereas CTLA4 can either directly inhibit TCR signaling in *cis* or out-compete CD28 for B7 ligands in *trans* (1–4). CD28-deficient humans have not yet been described, but CD28-deficient mice exhibit impaired T-cell proliferation (1, 5). Recently, heterozygous *CTLA4* loss-of-function mutations (*CTLA4*^wt/mut^) were identified in autoimmune lymphoproliferative syndrome type V patients (ALPS5; OMIM #616100) (6, 7). *CTLA4*^wt/mut^ T cells hyperproliferate *in vitro*, and inflamed ALPS5 patient organs exhibit gross lymphocytic infiltrates.

Herein, we describe for the first time the immunologic consequences of coexisting CD28 and CTLA4 haploinsufficiency in two unrelated human subjects. Their condition, a multi-organ inflammatory disease, appears superficially similar to ALPS5 but lacks distinctive lymphoproliferative features. Although our subjects’ CD28-haploinsufficient T cells display low *in vivo* proliferative histories and divide poorly *in vitro* to TCR-mediated activation, we demonstrate B7 blockade with CTLA4-Ig to be a highly effective anti-inflammatory monotherapy in this clinical context.

## Materials and Methods

### Human Samples

Peripheral blood mononuclear cell (PBMCs) were separated from peripheral blood samples of subjects and healthy donor (HD) controls *via* Ficoll-Paque centrifugation. The mean age of HDs was 26.3 years (range 14–50 years), and 40% were male. Demographic and clinical information on subjects are listed in Table S1 in Supplementary Material. Study protocols were approved by the institutional review boards of the Children’s Hospital of Philadelphia or the University of Messina. Written informed consent has been obtained from subjects for their cases to be published in this report.

### Flow Cytometry

The following antibodies were used for flow cytometric staining: anti-CD19 Pacific Blue, anti-CD14 Pacific Blue, anti-CD117 APC-Cy7, anti-CD4 APC-Cy7, anti-CD25 PE, anti-CD127 APC, anti-CD45RO AF-700, anti-CXCR5 Pacific Blue, anti-PD-1 PE-Cy7, anti-inducible T-cell costimulator (ICOS) BV510, anti-Ki67 Pe, and anti-CD28 PeDazzle (all from BioLegend) and anti-CD3 eFluor 605NC (eBioscience). Intracellular staining with anti-Foxp3 Alexa Fluor 488 (BioLegend), anti-CTLA4 BV786 (BD Bioscience), and anti-pAKT APC (eBioscience) was performed using the Foxp3/Transcription Factor Staining Buffer Set (eBioscience) in accordance with the manufacturer. Stained cells were then characterized by flow cytometry using a LSRFortessa (BD Bioscience).

### Cytokine Analysis

Interleukin-17A (IL-17A) and interleukin 22 (IL-22) concentrations in HD plasma and subject plasma obtained before and after 20 months of CTLA4-Ig therapy were determined using a LEGENDplex Th panel kit (BioLegend) in accordance with the manufacturer’s instructions.

### T-Cell Activation and Proliferation

CD4^+^CD45RO^−^ T cells were sorted using the MoFlo Astrios EQ (Beckman Coulter), carboxyfluorescein diacetate succinimidyl ester (CFSE) (Thermo Fisher Scientific) labeled and plated at 100,000 cells/well in a 96-well plate in RPMI 10% FBS either with 1 µg/ml phytohemagglutinin (PHA, Sigma) and 1 ng/ml rIL2 (R&D systems) or anti-CD2/CD3/CD28 coated beads [T regulatory cell (Treg) suppression inspector human, Miltenyi]. At 4 days, cellular proliferation was determined by flow cytometry. Anti-CD28 dose responses were determined by culturing PBMC or CFSE-labeled CD4^+^CD45RO^−^ T cells at 100,000 cells/well in a 96-well plate pre-coated with 1 µg/ml anti-CD3 (eBioscience) in the presence of various concentrations of anti-human CD28 (from 0.2 to 20 µg/ml; BD Biosciences). Phospho-AKT staining of CD4^+^ T cells was determined at 24 h by flow cytometry and CFSE dilution was assessed at 4 days.

### *In Vitro* Treg Suppression Assay

CD4^+^ T cells were pre-enriched using the MojoSort™ Human CD4 T Cell Isolation Kit (BioLegend). CD4^+^CD45RO^+^CD25^hi^CD127^lo/−^ Tregs and CD4^+^CD45RO^−^CD25^−^ Tresp cells were sorted and labeled with CFSE. Treg and Tresp cells were co-cultured at a 1:1 ratio in the presence of anti-CD2/CD3/CD28 coated beads. At 3.5–4.5 days, co-cultures were stained for viability with the LIVE/DEAD kit (Thermo Fisher Scientific), and the proliferation of viable Tresp cells was assessed using CFSE dilution.

### Immunohistochemistry

Tissue specimens were embedded in paraffin and fixed in formalin at the time of biopsy and held in storage. Prior to antibody staining, samples were deparaffinized and pre-treated with target retrieval solution (Dako, Santa Clara, CA, USA) for 15 min at boiling point. Slides were then cooled for 20 min before tissues were rinsed with buffer and subjected to antibody staining. Anti-CD3 (polyclonal, Dako) and anti-CD19 (Clone LE-CD19, Dako) both at a dilution of 1:200 were incubated with tissue sections at room temperature for 30 min. Sections were then mounted onto slides for visualization with bright field microscopy. For colonic biopsies, lymphoid cells infiltrating the glandular structures were enumerated across four representative regions and normalized to the number of gut epithelia in those same regions.

## Results

### Heterozygous Deletion of the *CD28/CTLA4/ICOS* Gene Cluster Is Associated With a Multi-Organ Inflammatory Disease

Our report focuses on two female subjects with intellectual disability and multi-organ inflammatory disease. Subject 1, now 20 years old, developed vitiligo universalis, thyroiditis, and lipodystrophy in her seventh year. At 12 years, she was diagnosed with anti-smooth muscle antibody-positive autoimmune hepatitis that was poorly responsive to azathioprine. Four years later, anti-GAD65 and anti-insulin antibody-positive insulin-dependent diabetes mellitus was recognized. Onset of chronic diarrhea at 17 years was associated with pan-enteropathic autoimmune enterocolitis. In her 18th year, she developed heart failure secondary to chronic pericarditis that progressed to tamponade physiology requiring a pericardiotomy (Figure S1 in Supplementary Material). Despite the unsuccessful application of various immunosuppressant therapies (azathioprine, rapamycin, colchicine, and corticosteroids), subject 1 exhibited an unremarkable infectious history. Laboratory assessments of protective immunity were generally unrevealing. She possessed normal serum IgG (1,320 mg/dl, nml 635–1,775) and IgM concentrations (110 mg/dl, nml 71–237) but a lower IgA concentration (44 mg/dl, nml 70–486). She generated protective vaccine titers to tetanus, diphtheria, and pneumococcus (Table S1 in Supplementary Material).

Subject 2, now 6 years old, also presented in infancy with neurodevelopmental delay and feeding difficulties. At that time, her skin was affected by a severe form of atopic dermatitis that evolved over her childhood into lichen sclerosis. At 15 months of age, subject 2 developed autoimmune urticaria and chronic diarrhea-associated enterocolitis with an associated recto-perineal fistula requiring surgical correction. At 6 years of age, subject 2 was diagnosed with anti-thyroperoxidase antibody-positive autoimmune thyroiditis. Despite a history of respiratory tract infections, assessments of subject 2 humoral immune function revealed normal IgG (710 mg/dl, nL 633–1,280) and IgM serum levels (49 mg/dl, 48–207) but a low IgA level (5 mg/dl, 33–202, Table S1 in Supplementary Material).

**Figure 1:**
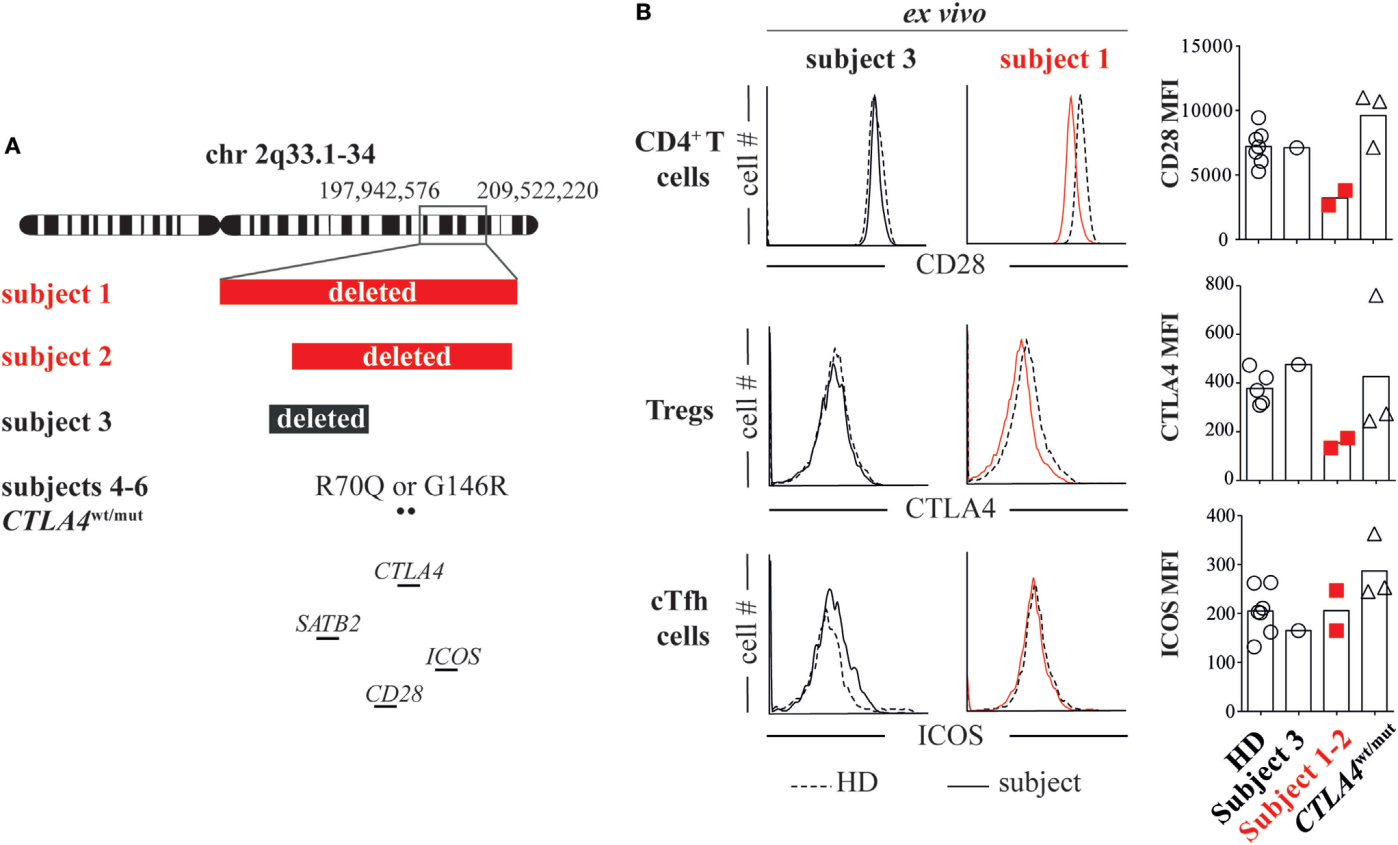
Heterozygous loss of the *CD28*/*CTLA4*/*ICOS* gene cluster decreases CD28 and cytotoxic T-lymphocyte-associated protein 4 (CTLA4) but not inducible T-cell costimulator (ICOS) expression. **(A)** Chromosome 2 breakpoints and mutation locations for subjects 1 through 6 are depicted. **(B)** *Ex vivo* CD4^+^ T cell CD28 expression, T regulatory cell (Treg) (CD127^low^CD25^high^) CTLA4 expression and ICOS expression on cTfh cells (CXCR5^+^PD1^+^) were assessed in subject 1 (red line), subject 3 (black line), and healthy donor (HD, dashed line) blood samples by flow cytometry. CD28, ICOS, and CTLA4 average mean fluorescence intensities (MFIs) from subjects and HDs are summarized (right).

To determine if there was a genetic basis for their inflammatory diseases, we analyzed subject 1’s DNA using whole exome sequencing, which was unrevealing, and using chromosomal microarray, which identified a *de novo* heterozygous 11.6 Mb chromosome 2q33.1-q34 deletion (197,942,576–209,522,220; GRCh37/hg19). Several potentially pathologic genes within the deleted region were of interest including the intellectual disability gene *SATB2* and the *CD28*/*CTLA4*/*ICOS* immune gene cluster (Figure 1A). Using a chromosomal microarray, we identified a more discrete *de novo* 8.3 Mb chromosome 2q33.1-q34 deletion (200,742,119–209,062,408), also encompassing *SATB2* and the *CD28*/*CTLA4*/*ICOS* gene cluster, in subject 2 (Figure 1A).

### Heterozygous Deletion of *CD28/CTLA4/ICOS* Diminishes *Ex Vivo* Expression of CD28 and CTLA4 but Not ICOS

To investigate if heterozygous loss of *CD28*/*CTLA4*/*ICOS* alters protein expression, we compared CD28, CTLA4, and ICOS mean fluorescence intensities (MFIs) on subject 1/subject 2 T-cell subsets to MFIs on HD cells and also cells from a separate control subject (subject 3). Subject 3 is an intellectually disabled 9-year-old female with a *de novo* heterozygous 5 Mb 2q33.1 deletion encompassing *SATB2* but not *CD28*/*CTLA4*/*ICOS* (199,631,959–204,602,266; GRCh37/hg19, Figure 1A; Table S1 in Supplementary Material) (8). She is immune competent and has not experienced inflammatory diseases. In agreement with previously published T-cell phenotypes from unaffected parents of ICOS-deficient patients (9) and consistent normal circulating CXCR5^+^PD-1^+^ T follicular helper (cTfh) cell frequencies (Table S1 in Supplementary Material), we found ICOS expression on subject 1/subject 2 cTfh cells, Tregs and naïve T cells to be indistinguishable from HD/subject 3 T-cell subsets (Figure 1B; Figure S2 in Supplementary Material). By contrast, CD28 and CTLA4 MFIs on subject 1/subject 2 CD4^+^ T cells and CD25^hi^CD127^−^ Tregs were, respectively, half of HD/subject 3 counterpart cell MFIs (Figure 1B). Hence, heterozygous deletion of the *CD28*/*CTLA4*/*ICOS* gene cluster diminishes expression of CD28 and CTLA4 but not ICOS.

### CD28 Haploinsufficient T Cells Are Hypoproliferative

**Figure 2:**
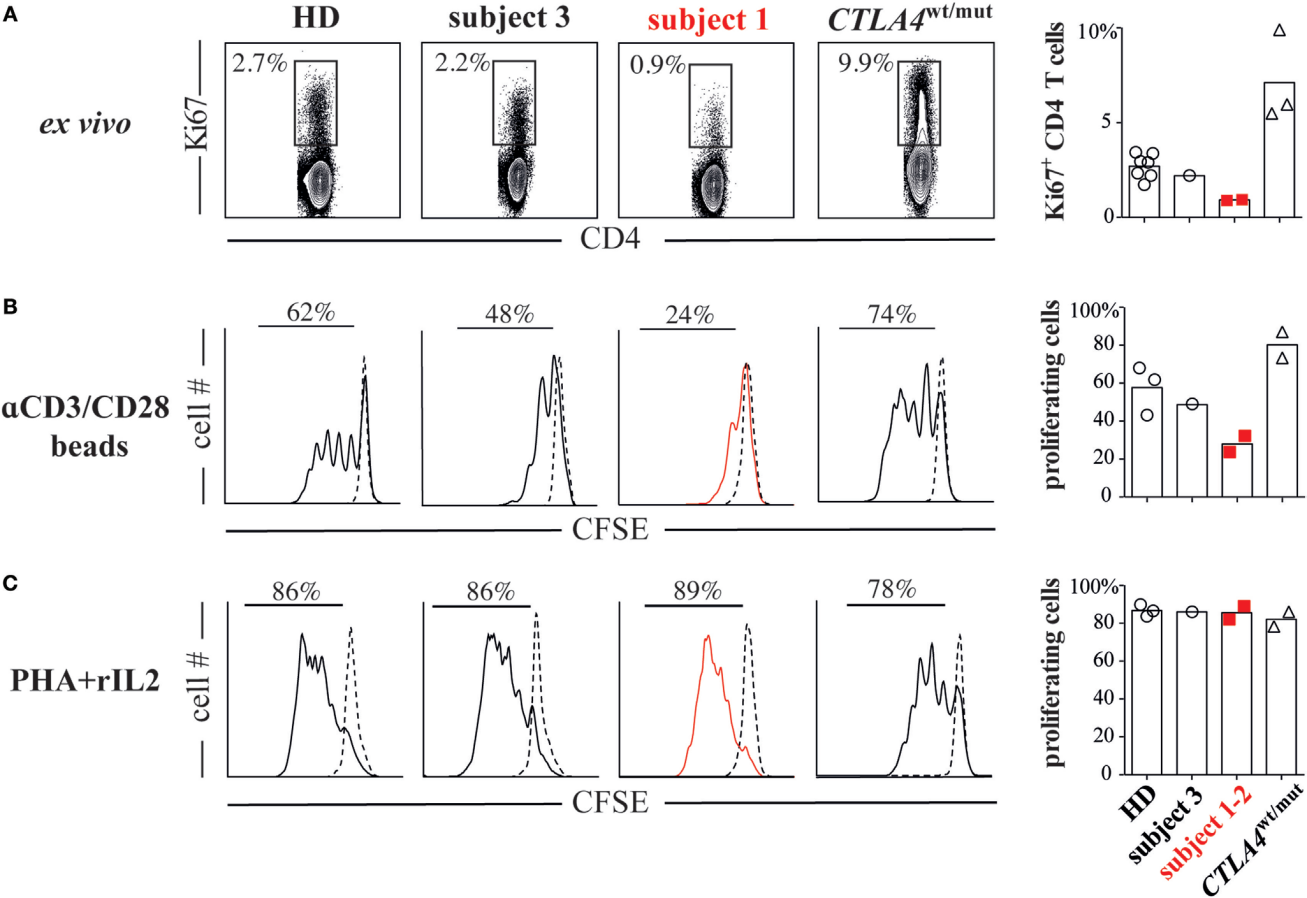
CD28 haploinsufficiency is associated with T-cell hypoproliferation. **(A)** Subject 1/subject 2 display decreased frequencies of Ki67^+^ CD4^+^ T cells. **(B)** CFSE dilutions in sorted naïve T cells (CD4^+^CD45RO^−^) from subjects and healthy donor (HDs) cultured for 4 days with anti-CD2/CD3/CD28 coated beads or **(C)** phytohemagglutinin (PHA, 1 µg/ml) and recombinant interleukin 2 (rIL-2, 1 ng/ml) are displayed.

To identify the functional consequences of reduced CD28 expression, we compared subject 1/subject 2 T-cell proliferative responses against HD/subject 3 cells and also against cells from three additional subjects (subjects 4 through 6) with heterozygous ALPS5-associated *CTLA4* mutations (*CTLA4*^wt/mut^, Table S1 in Supplementary Material). Although CTLA4 expression varied significantly on *CTLA4*^wt/mut^ cells, CD28 and ICOS expression did not and was indistinguishable from HD control cells (Figure 1B). As previously reported (6, 10), *CTLA4*^wt/mut^ CD4^+^ T cells skewed toward a memory phenotype and an increased frequency expressed the cell-cycle marker Ki67 (5–9%) compared with HD/subject 3 counterpart cells (2–3%). By contrast, less than 1% of subject 1 and subject 2 CD4^+^ T cells were Ki67^+^, suggesting a recent history of hypoproliferation (Figure 2A). To determine if subject 1/subject 2 proliferative responses were also reduced *in vitro*, we sorted their naïve T cells and stimulated them with anti-CD3 and anti-CD28 antibodies. After 4 days, only 24% of subject 1 and 32% of subject 2 T cells had divided, a proportion roughly half that of identically stimulated HD (58%) and subject 3 T cells (48%). By contrast, 80% of *CTLA4*^wt/mut^ naïve T cells divided by culture day 4, a finding that agreed with previously published reports (Figure 2B) (6, 7). Increasing anti-CD28 concentrations improved subject 1 T-cell division somewhat but did not normalize AKT phosphorylation, a downstream indicator of CD28/TCR activity (Figure S3 in Supplementary Material). Notably, when stimulated with PHA and IL-2, subject 1/subject 2 T cells proliferated similar to HD controls (Figure 2C). Hence, CD28 haploinsufficiency imparts a pathway-specific T-cell proliferation defect.

### Tregs From CD28/CTLA4 Haploinsufficient Patients Are Quantitatively and Qualitatively Diminished

**Figure 3:**
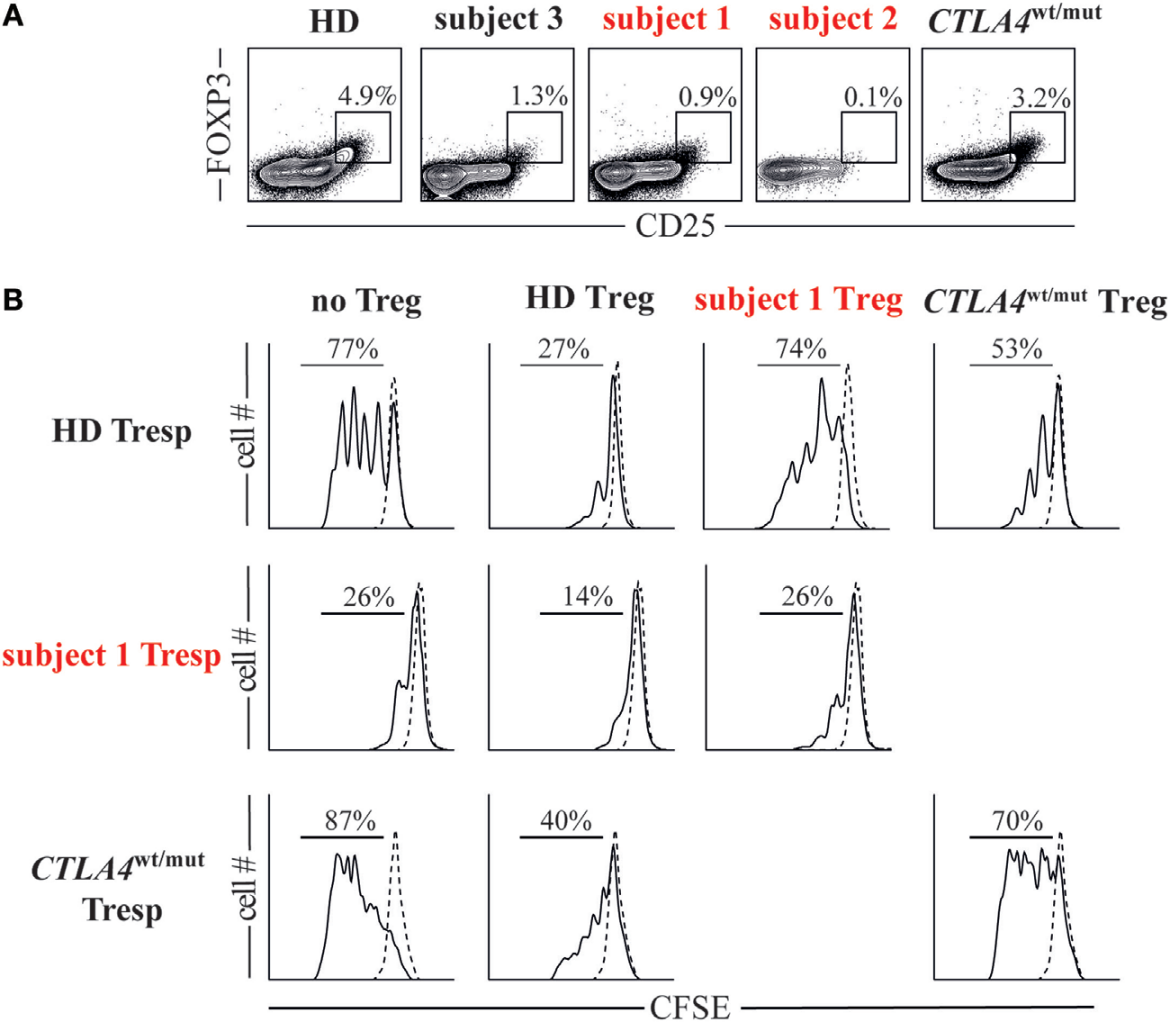
Cytotoxic T-lymphocyte-associated protein 4 (CTLA4)/CD28 haploinsufficiency is associated with quantitative and qualitative T regulatory cell (Treg) defects. **(A)** Frequencies of Foxp3^+^CD25^hi^ Tregs in subjects and a representative healthy donor (HD) are displayed. **(B)** Histograms display day 4 cell proliferation of unstimulated (dotted line) and anti-CD2/CD3/CD28 stimulated (black line) T responder (Tresp) cells cultured with or without autologous or heterologous CD25^hi^CD127^lo^ Tregs in a 1:1 Tresp to Treg ratio.

To assess the suppressive potential of subject 1/subject 2 FOXP3^+^ Tregs, we first determined their frequencies in peripheral blood. Tregs comprised only 1% of subject 1 CD4^+^ T cells and 0.1% of subject 2 CD4^+^ T cells, considerably lower values than HDs (2.8–7.8%) and *CTLA4*^wt/mut^ subjects (0.9–3.2%, Figure 3A). We then assessed the ability of subject 1 Tregs to suppress proliferation of naïve T responder cells (Tresp) when cultured in a 1:1 ratio. At culture day 4, subject 1 Tregs suppressed heterologous HD Tresp proliferation as poorly as *CTLA4*^wt/mut^ Tregs, which were previously reported to exhibit this defect (Figure 3B) (6, 7). Thus, CTLA4 haploinsufficiency-associated Treg defects are balanced *in vitro* by CD28 haploinsufficiency-associated Tresp cell hypoproliferation.

### Inflamed Subject 1 Tissues Lack T-Cell and B-Cell Infiltrates

**Figure 4:**
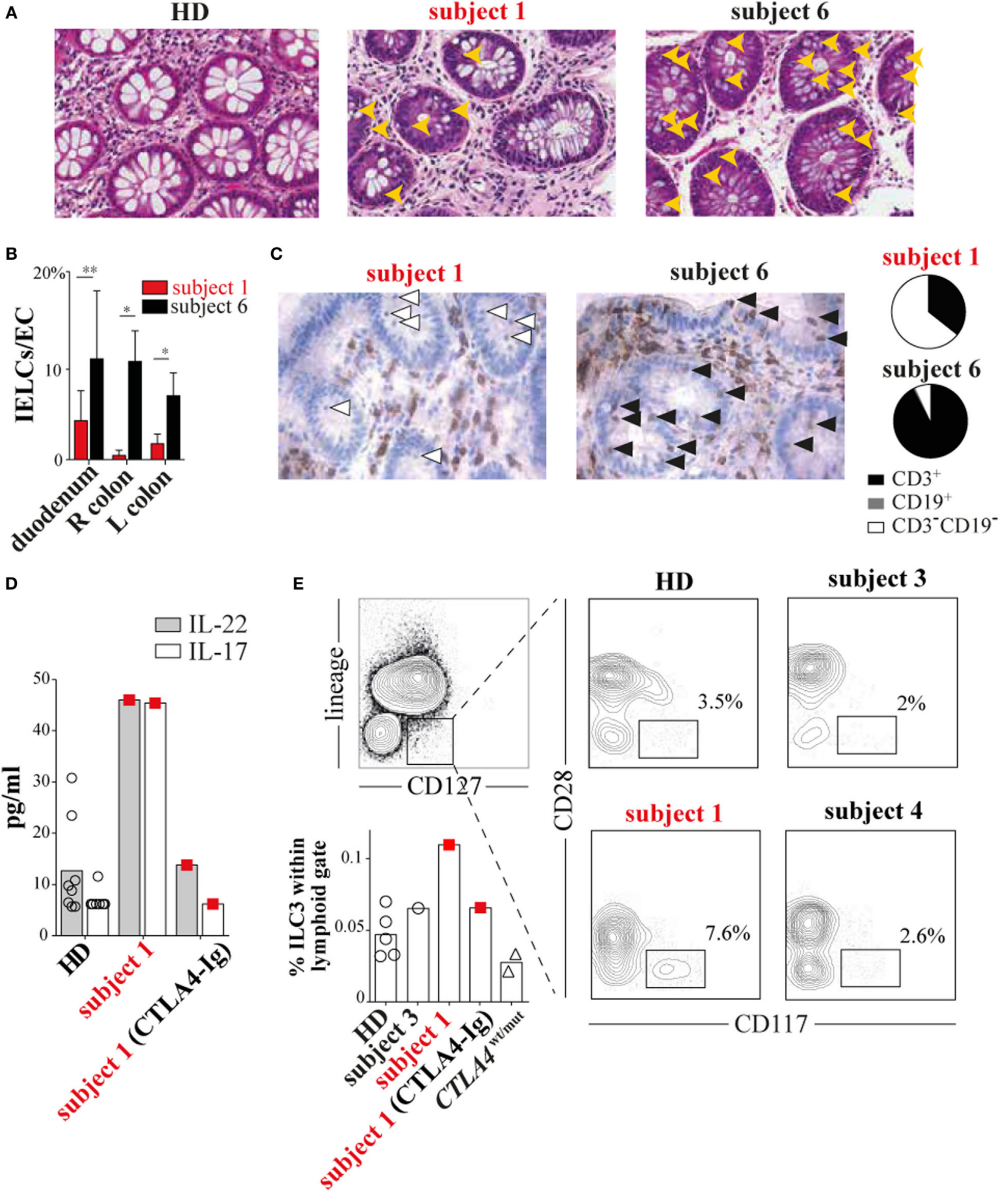
Subject 1 displays scant T-cell gut infiltrates with increased circulating type 3 innate lymphoid cells (ILC3s) and ILC3 cytokines. **(A)** Hematoxylin and eosin staining of healthy donor (HD), subject 1 and subject 6 colonic biopsies with intraepithelial lymphoid cells (IELCs) indicated (gold arrowheads). 40× original magnification for all images. **(B)** Average frequencies of IELCs per gland epithelial cell from subjects’ duodenum, right (R) colon, and left (L) colon biopsies are displayed (*P < 0.029, **P < 0.04, Mann–Whitney *U* test). **(C)** Anti-CD3 immunohistochemical staining (left) identifies T cells (black arrowheads) and non-T cells (white arrowheads). Pie charts (right) depict the proportions of different infiltrating lymphoid cell populations in subject 1 and subject 6 colonic biopsies. Total lymphoid cells counted per biopsy are *n* = 57 and *n* = 72, respectively. **(D)** Subject 1 plasma interleukin 22 (IL-22) and interleukin-17A (IL-17A) concentrations and **(E)** circulating ILC3 (CD3^−^CD19^−^CD14^−^CD11c^−^CD127^+^CD117^+^CD28^−^) frequencies are elevated and decrease after 20 months of cytotoxic T-lymphocyte-associated protein 4 (CTLA4)-Ig therapy.

During the course of her many inflammatory diseases, subject 1 was multiply biopsied. To determine how T-cell defects we observed *in vitro* contributed to inflammatory pathology *in vivo*, we analyzed subject 1 colon, pericardium, liver, and duodenal tissues obtained during periods of active disease. Tissue damage was prominent in each sample, but lymphoid infiltrates were scant (Figure 4A; Figures S1, S4, and S5 in Supplementary Material). This pattern was especially striking in contrast to tissues from *CTLA4*^wt/mut^ patients like subject 6 (6, 7). Although subject 1 and subject 6 gastrointestinal biopsies both exhibited pathologic surface epithelium attenuation, goblet cell loss, and increased crypt epithelium apoptosis, only subject 6 tissues exhibited prominent intraepithelial lymphoid cell (IELC) infiltrates, a common feature of autoimmune enteropathies (11) (Figure 4A, gold arrowheads). In fact, we found IELC frequencies in subject 6 duodenum, left colon and right colon to be 2.5×, 4×, and 20× greater than subject 1 biopsies from the same sites (*P* < 0.029, *P* < 0.029, and *P* < 0.04, Figure 4B). Immunohistochemical staining revealed that while most IELCs in subject 6 gastrointestinal biopsies were T cells (93%) (Figure 4C, black arrowheads), most (64%) subject 1 IELCs stained negative for CD3 and CD19 lineage markers (Figure 4C, white arrowheads and Figure S6 in Supplementary Material). Inflamed subject 2 tissues were not biopsied and therefore were unavailable for analysis.

### Increased Circulating Type 3 Innate Lymphoid Cells (ILC3s) and ILC3 Cytokines Are Modulated by CTLA-Ig Therapy

Based upon encouraging reports describing improvement of ALPS5 enteropathy with CTLA4-Ig treatment (12, 13) and since other therapies had been ineffective, we began subject 1 on a trial of CTLA4-Ig. CTLA4-Ig monotherapy proved remarkably effective in treating subject 1 enterocolitis, hepatitis, and pericarditis, although its use was associated with progressive antibody deficiency. When comparing multiplex cytokine profiles of subject 1 plasma from before and during CTLA4-Ig therapy, the most dramatic differences were decreases in IL-22/IL-17A concentrations (Figure 4D).

Recently, ILCs, which lack antigen-specific receptors and traditional lineage markers (CD3, CD19, CD14, etc.), have emerged as potent mucosal cytokines producers, including IL-22 and IL17 (14). To determine if changes in circulating ILCs correlated with her inflammatory symptoms, we compared ILC frequencies in subject 1’s blood before CTLA4-Ig to frequencies in blood from HDs and subjects 3 through 6. All were similar (Figure S7 in Supplementary Material). ILCs can be further divided into types 1 through 3 on the basis of cytokine secretion signatures or by CD28 expression; CD28 is highly expressed by ILC1s and ILC2s but not ILC3s (15). Using a CD28 and CD117 gating strategy, we determined that ILC3s, the primary secretors of IL-22 and IL-17, were present at twice the frequency in subject 1 blood samples taken before CTLA4-Ig therapy than were present in HD samples (Figure 4E). With CTLA4-Ig, subject 1 ILC3 frequencies dropped precipitously and became indistinguishable from controls.

## Discussion

Herein, we describe two phenotypically similar subjects each with heterozygous loss of the CD28/CTLA4/ICOS gene cluster. Like *CTLA4*^wt/mut^ ALPS5 patients, our subjects experienced multi-organ inflammation and exhibited a Treg suppressive defect. Yet, while ALPS5 patients typically display normal or only slightly reduced Treg numbers (6, 7), we found subject 1 Treg frequencies to be significantly reduced and subject 2’s Tregs to be nearly absent. Such scarcity parallels Treg-specific *CD28* conditional knockout mice that experience multi-organ inflammatory disease because they fail to numerically balance suppressor and effector T cells during immune responses (16). Yet, unlike this mouse model, our subjects’ Tregs intrinsically lack suppressive capacity due to CTLA4 haploinsufficiency and our subjects’ effector T cells hypoproliferated to traditional TCR-focused activation due to CD28 haploinsufficiency. As T-cell hyperproliferation and related lymphocytic organ infiltration are the *sine qua non* of ALPS5 pathology, we conclude the inflammatory process affecting subject 1 and subject 2 is distinct from those affecting *CTLA4*^wt/mut^ patients.

Instead of T-cell expansion in subject 1, we found that coexisting CD28 and CTLA4-haploinsufficiency correlated with a dramatic increase in circulating ILC3s and ILC3 cytokines. We speculate that because ILC3s do not express CD28, they may enjoy, in our subjects, a competitive advantage over ILC1s and ILC2s which potentially rely upon CD28 for homeostasis. As ILC3s have been previously shown to mediate mouse colitis and to be increased in the inflamed intestines of Crohn’s disease patients (14), expansion of these cells may explain subject 1’s enterocolitis and their contraction, its resolution. Currently, the prevailing view is that CTLA4-Ig dampens inflammation by out-competing CD28 expressed on T cells for B7 molecules (17), but given low CD28 expression and reduced Tresp cell proliferation, this mechanism does not adequately explain subject 1’s dramatic CTAL4-Ig response. One clue, in our specific context, may come from previous work demonstrating that CTLA4-Ig prolongs allograft survival in *CD28*/*CTLA4* double knockout mice (18) and experiments that reveal CTLA4-Ig, but not CD28 blockade, inhibits T-dependent alloantibody production in rats (19). These related phenomena, first observed in animals and now in our CTLA4/CD28 haploinsufficient subjects, predict that a stimulatory B7 receptor(s) other than CD28 exists and that it is expressed by ILC3s.

## Ethics Statement

This study was carried out in accordance with the recommendations and approved by the institutional review boards of the Children’s Hospital of Philadelphia and the University of Messina. All subjects gave written informed consent in accordance with the Declaration of Helsinki.

## Author Contributions

CC, MT, BN, AK, and NR performed experiments and analyzed data. BN and TB performed histological analyses. KS, SB, EZ, and NR provided patient samples. MK, SL, AN, and EZ genotyped subjects. CC and NR were responsible for study design and wrote the manuscript. All the authors reviewed the manuscript and provided scientific input.

## Conflict of Interest Statement

The authors declare that the research was conducted in the absence of any commercial or financial relationships that could be construed as a potential conflict of interest.
