# Supplementary material for: Cytotoxic T-Lymphocyte-Associated Protein 4 Haploinsufficiency-Associated Inflammation Can Occur Independently of T-Cell Hyperproliferation
Source: Front Immunol. 2018 Jul 24;9:1715. doi: 10.3389/fimmu.2018.01715 (PMC6066513; doi:10.3389/fimmu.2018.01715)
Supplement: Supplementary file 1 [file Data_Sheet_1.PDF]

Table S1. Subject demographic, genotype, immune phenotype and clinical information

| Subject ID | S1 | S2 | S3 | S4 | S5 | S6 |
|---|---|---|---|---|---|---|
| Age (years) | 20 | 6 | 9 | 21 | 49 | 28 |
| Sex | F | F | F | M | F | F |
| Genotype | heterozygous deletion Chr2q33.1-34 (12 Mb) | heterozygous deletion Chr2q33.1-34 (8.3 Mb) | heterozygous deletion Chr2q33.1 (2.4 Mb) | heterozygous mutation CTLA4 p.R70Q | heterozygous mutation CTLA4 p.R70Q | heterozygous mutation CTLA4 p.G146R |
| Inflammatory diseases | DM1, EC, H, LD, PC, T, VU | AIU, EC, T | none | ES | none | A, CLIP, ES, R |
| $CD4^+$ T cell frequency† | 24.7% | 43.6% | 43.8% | 34.2% | 45.1% | 36.2% |
| $CD4^+$ T cell naïve to memory ratio | 1:0.5 | 1:0.2 | 1:0.3 | 1:8 | 1:0.9 | 1:2 |
| cTfh frequency* | 14% | 8.6% | 8.3% | 13.5% | 3.5% | 10% |
| $CD8^+$ T cell frequency† | 17.8% | 18.9% | 19% | 28% | 10.7% | 18.1% |
| $CD8^+$ T cell naïve to memory ratio | 1:0.1 | 1:0.01 | 1:0.1 | 1:0.8 | 1:2 | 1:0.3 |
| IgG (mg/dL) | 1320 | 710 | 721 | 379 (L) | ND | 361 (L) |
| IgA (mg/dL) | 44 (L) | < 5 (L) | 63 | 28 (L) | ND | < 6 (L) |
| IgM (mg/dL) | 110 | 49 | 127 | 10 (L) | ND | 25 (L) |
| PCV23 vaccine response | 9/14 protective | ND | 11/14 protective | 0/14 protective | ND | 0/14 protective |

A, arthritis; AIU, autoimmune urticaria; CLIP, chronic lymphoid interstitial pneumonitis; DM1, diabetes mellitus type 1; EC, enterocolitis; ES, Evans Syndrome; F, female; H, hepatitis; L, below normal range; LD, lipodystrophy; M, male; ND, not done; PC, pericarditis; PCV, pneumococcal conjugate vaccine; R, retinitis; T, thyroiditis; VU, vitiligo universalis

† frequencies determined by the percentage of indicated cells among all lymphocytes

*frequencies determined by the percentage of $CXCR5^+PD1^{hi}$ cells among all memory $CD4^+$ cells
