# Supplementary material for: Cytotoxic T-Lymphocyte-Associated Protein 4 Haploinsufficiency-Associated Inflammation Can Occur Independently of T-Cell Hyperproliferation
Source: Front Immunol. 2018 Jul 24;9:1715. doi: 10.3389/fimmu.2018.01715 (PMC6066513; doi:10.3389/fimmu.2018.01715)
Supplement: Supplementary file 2 [file Data_Sheet_2.PDF]

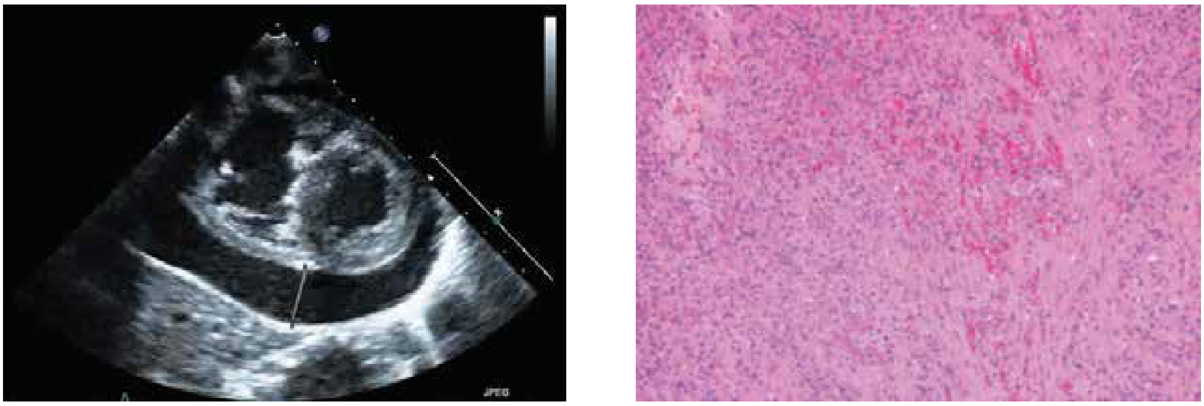

Figure S1. Inflammatory pericarditis in subject 1. A large pericardial effusion is captured on a parasternal short-axis view of a transthoracic ultrasound (left). A pericardial window biopsy obtained during illness stained with hematoxylin and eosin demonstrates chronic fibrosis, a neutrophilic infiltrate and scant lymphocytes. 20x original magnification.

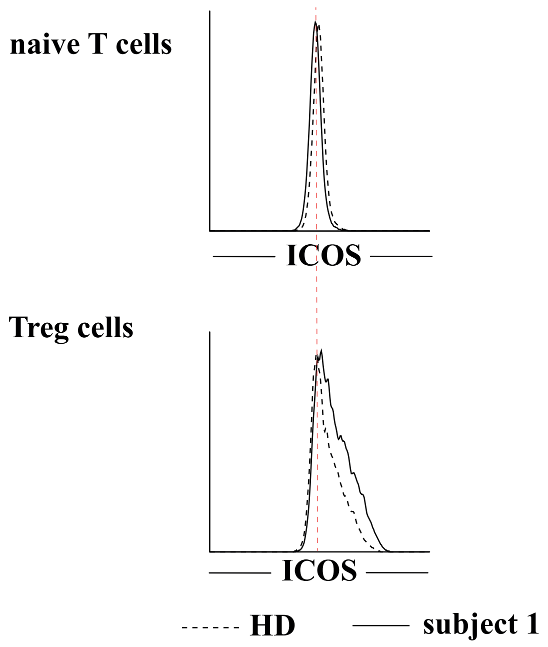

Figure S2. Heterozygous loss of *ICOS* does not altered ICOS expression. *Ex vivo* ICOS expression on naïve CD4^{+} T cells (CD4^{+}CD45RO^{-}) and Tregs (CD127^{low}CD25^{high}) in subject 1 (black line) and a healthy donor (dashed line) as determined by FACS.

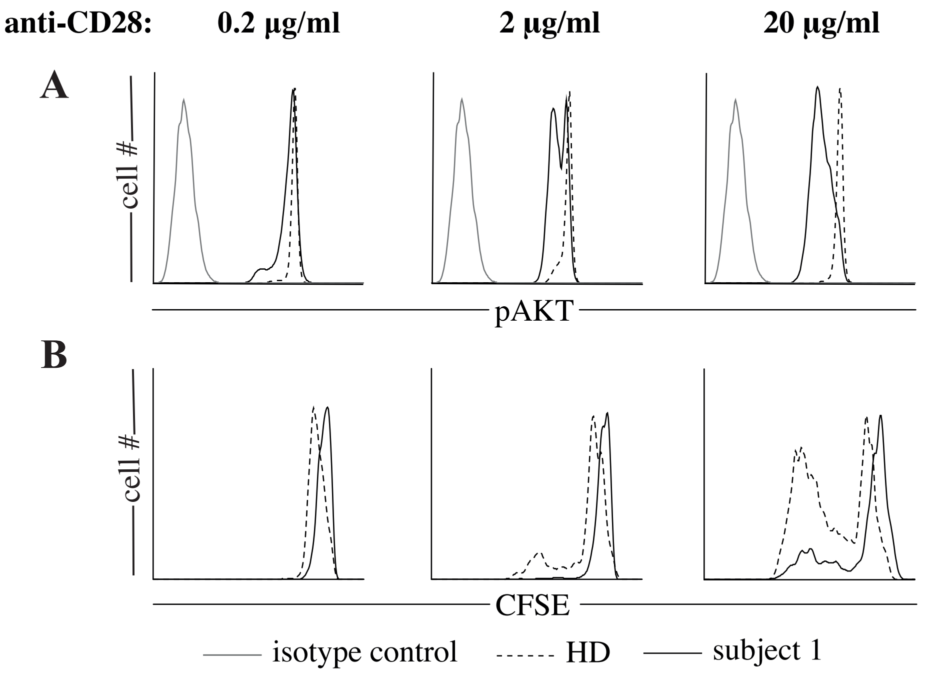

Figure S3. Subject 1 T cells respond to super physiologic anti-CD28 agonism. (**A**) Subject 1 CD4^{+} T cells exhibit less phospho-AKT at 24 hours and (**B**) less proliferation by CFSE dilution at four days than control cells. Cells were cultured in anti-CD3 (1µg/ml) and increasing concentrations of anti-CD28.

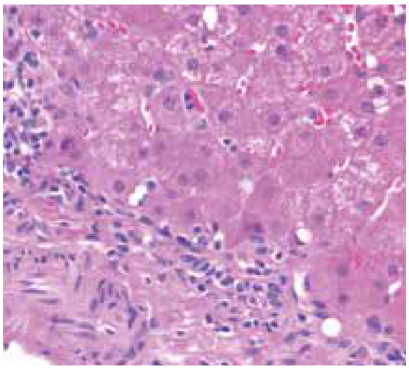

Figure S4. Hepatitis in subject 1. Hematoxylin and eosin stained liver biopsy tissue displays fibrosis and scant-centric infiltration. 20x original magnification.

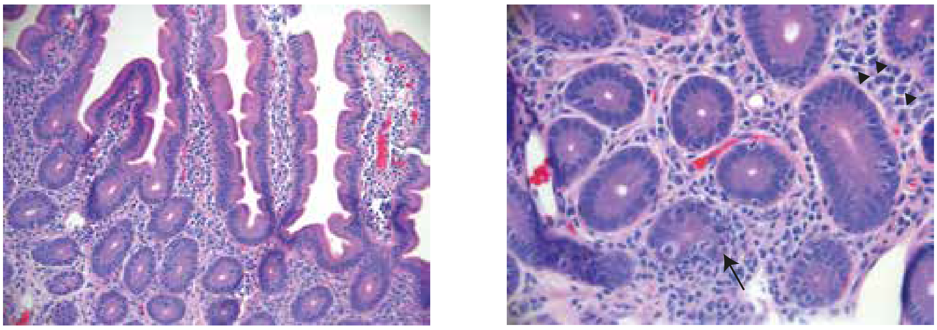

Figure S5. Duodenitis in subject 1. Hematoxylin and eosin stained duodenal biopsy tissue displays a loss of goblet cells, increased apoptosis within glands but few intraepithelial lymphoid cells. Plasma cells are highlighted with arrowheads; an apoptotic cell is highlighted with an arrow. 20x original magnification.

subject 1 subject 6

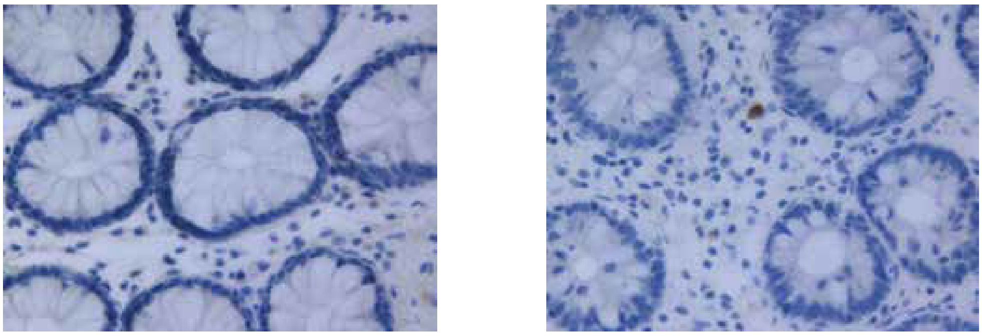

Figure S6. B cells do not infiltrate the colonic intraepithelial space in CTLA4 haploinsufficiency-associated colitis. Immunohistochemical staining of subject 1 and subject 6 colonic biopsies show few or no infiltration $CD19^+$ cells in the intraepithelial space. 40x magnification.

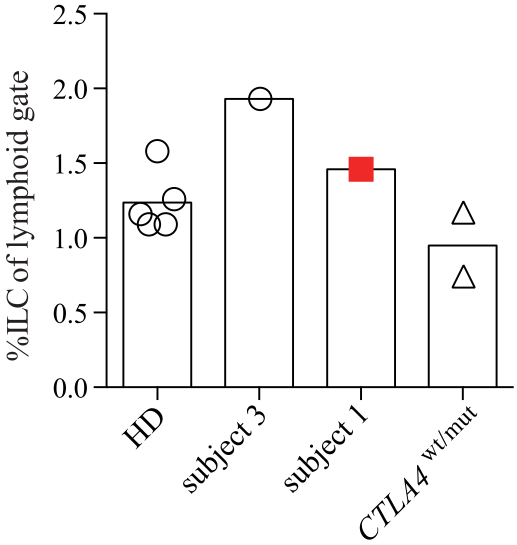

Figures S7. Circulating innate lymphoid cell (ILC) frequencies are not affected by CTLA4 or CTLA4/CD28 haploinsufficiency. ILC ($CD3^{-}CD19^{-}CD14^{-}CD11c^{-}CD127^{+}$) frequencies are displayed for healthy donors and subjects as a percentage of all cells in the lymphoid gate.
